# A systematic review and meta-analysis on the association of serum and tumor tissue iron and risk of breast cancer

**DOI:** 10.22088/cjim.11.1.1

**Published:** 2020

**Authors:** Akram Sanagoo, Faezeh Kiani, Marzieh Saei Gharenaz, Fatemeh Sayehmiri, Fatemeh Koohi, Leila Jouybari, Majjid Dousti

**Affiliations:** 1 Nursing Research Center, Golestan University of Medical Sciences, Gorgan, Iran; 2 Student Research Committee, Ilam University of Medical Sciences, Ilam, Iran; 3 Students Research Committee, Shahid Beheshti University of Medical Sciences, Tehran, Iran; 4 Student Research Committee, Proteomics Research Center, Shahid Beheshti University of Medical Sciences, Tehran, Iran; 5 Social Determinants of Health Research Center, Birjand University of Medical Sciences, Birjand, Iran; 6 Faculty of Medicine, Ilam University of Medical Sciences, Ilam, Iran

**Keywords:** Breast Cancer, Neoplasm, trace elements, Iron, Meta-analysis

## Abstract

**Background:**

Some studies have investigated the effects of iron on breast carcinogenesis and reported different findings about the association between Fe and breast cancer risk. This study was conducted to estimate this effect using meta-analysis method.

**Methods:**

A total of 20 articles published between 1984 and 2017 worldwide were selected through searching PubMed, Scopus, Embase, Web of Science, and Cochrane Library. Keywords such Breast Cancer, Neoplasm, Trace elements, Iron, Breast tissue concentration, Plasma concentration, Scalp hair concentration, toenail concentration and their combination were used in the search.

**Results:**

The total number of participants was 4,110 individuals comprising 1,624 patients with breast cancer and 2,486 healthy subjects. Fe concentration was measured in the various subgroups in both case and control groups. There were significant correlations between Fe concentration and breast cancer in breast tissue subgroup (SMD: 0.67 [95% CI: 0.17 to 1.17; P=0.009]). Whereas, there was no meaningful difference in Fe status between women with and without breast cancer related to scalp hair and plasma subgroups; (SMD: -3.74 [95% CI: -7.58 to 0.10; P=0.056] and (SMD:-1.14[95% CI: -2.30 to 0.03; P=0.055], respectively.

**Conclusion:**

The present meta-analysis indicated a positive and straight association between iron concentrations and risk of breast cancer but because of high heterogeneity we recommend more accurate future studies.

Breast cancer has the most cancer incidence in women and also the second most common global cancer (1, 2). It represents the fifth cause of cancer deaths and the first cause of cancer death in women around the world (3, 4). Although, mortality rate of breast cancer has decreased in developed regions but it still represents the first cause of death due to cancer in less developed regions and the second cause in developed regions (5, 6). So, understanding the etiology and factors involved in breast carcinogenesis can contribute to treatment options (7). Some studies have shown that not only inherited genetic factors increase the breast cancer risk; but also, lifestyle and environment are important risk factors for breast cancer (8, 9). Numerous studies have established involvement of metallic compounds like trace elements in the development of breast cancer (10-12). Trace elements elicit several biological functions; participate in the synthesis of hormones and vitamins, regulate gene expression, modulate cell membrane permeability and take part in electron transport (13); also are involved in hormonal and enzymatic function because they compete with other metals for potential interaction sites (14). 

These elements can be essential and benefit in small concentrations or toxic and carcinogenic, when taken in excess (15). Researchers demonstrated that trace elements are able to influence breast carcinogenesis in multiple ways; they act as estrogen disruptors and active estrogen signaling which a network of important biological mechanisms in the carcinogenesis process like, proliferation and migration of breast cancer cells trigger (10, 16).

One of them is Fe- in carcinogenesis of breast cancer. Iron works as a structural and functional cofactor for various proteins and enzymes, a prosthetic group in many enzymes and an important constituent of succinate dehydrogenase as well as a part of the hem of hemoglobin, myoglobin, and the cytochromes (15, 17). It is a key element in different biochemical cell processes and the integrity of various cell apparatus, and also involved in many physiological functions like oxygen transportation, oxidative phosphorylation and xenobiotic metabolism (17). However, high Fe intake can have carcinogenic effects and leads to development of cancer due to the fact that Fe is crucial for regulating of cell growth (18). Furthermore, iron may act as a catalyst, exert toxicity by generating highly toxic molecules and involve in the repress of host defense cells (19). Due to the high incidence of breast carcinomas and its mortality and morbidity rate in the world, prevention planning and treatment this disease seemed necessary. The management of risk factors is an appropriate option for prevention of non-communicable diseases, thus determining the risk factors involved in breast carcinogenesis can contribute to treatment options. 

There are many studies with different findings concerning the influence of Fe in the incidence of breast cancer; and lack of systematic review and meta-analysis in this area to provide an overall and valid result, conducting a meta-analysis is important; because a meta-analysis study leads to a large sample size and overall resolution. The aim of this study was to investigate the influence of Fe on breast cancer by reviewing the available studies.

## Methods

**Study selection: **We performed a literature search with citations in the Scopus, PubMed, Web of Science, Embase, and Cochrane Library databases for case-control and cross sectional published between 1984 and 2017. The search strategy was done via keywords: Breast Cancer, Trace elements, Iron, Breast tissue concentration, Plasma concentration, Scalp hair concentration, toenail concentration and their combination. The search scope was developed using the wildcard symbol ‘*’ and an advanced search was performed with the combination of words or phrases using Boolean operators (‘AND’, ‘OR’, ‘NOT’). The titles from searching were scanned to be appropriated for inclusion in the study. Furthermore, the lists of reference from all related reviews and main articles were manually searched for more references. 

**Inclusion and exclusion criteria: **All studies that analyzed Fe levels in breast Cancer patients were reviewed. Each screened study for inclusion in final analysis must present the data on the relation between Fe levels and breast cancer risk by measuring this element concentration in any of three types of sample specimens: serum, breast tissue and hair. Study was limited to conduct articles in humans. We excluded studies if they were duplicate publications or that were meta-analyses or systematic considerations; if they perform on non-human creatures (i.e. animal studies); if they presented insufficient data; and if published in languages other than English. We searched gray literature as far as possible in search engineers, but access to all of the gray literature sours was not possible. 

**Data extraction: **The extracted data for all studies (case- control and cross sectional) include the first author, year of publication, sample size, sample age, location, iron concentration, mean difference, type of sample specimens, and iron screening method (figure 1-study flowchart). Two of the authors independently extracted information from each article and compared findings. In which cases, the results were discordant, papers were reviewed jointly and discrepancies between researchers were resolved via group discussions. 

**Evaluation of the quality of selected studies: **In this study, Newcastle–Ottawa Scale checklist was used to assess the quality of studies (table 1). The NOS (Newcastle–Ottawa Scale) ranges from zero to nine stars. Selected papers were ranked in three groups according to NOS quality assessment: 1- low quality (up to 3 stars), 2- medium quality (4-6 stars) and 3- high quality (more than seven stars).

The criteria considered to measure bias in the authors' intended studies included: reference to the time and place of the study, describing exit and entry criteria of the participants, how to measure the variables, what statistical methods used and preparing reports on standard deviation or confidence intervals of the estimates.

**Table 1:** Quality assessment of included articles according to the Newcastle–Ottawa Scale checklist

	**Selection**				**Comparability**		**Exposure**		
Author (References)	1	2	3	4	A	B	1	2	3
Rizk.Sh.L (23)		*			*			*	*
N.Drake II.E (24)		*		*	*		*	*	
Singh.V (25)		*			*		*	*	
Geraki.K (20)		*			*			*	
Wen Kuo.HW (21)	*	*		*	*	*		*	*
Ionescu.J.G (39)		*			*	*			
Cui.Y (27)	*	*		*	*		*	*	
Ebrahim.A.M (28)		*			*	*		*	*
Magalhaes.T (29)		*			*	*		*	*
Arinola, O. G (30)	*	*			*	*	*	*	
Joo.N.S (22)	*	*		*	*	*	*	*	
Pasha. Q (31)	*	*			*	*		*	
Feng.J (32)	*	*		*	*	*	*	*	
Silva.M.P (33)	*	*			*	*		*	
Rehman.S (34)	*	*						*	
Pavithra. V(35)	*				*		*	*	
Karki.K (36)	*	*		*	*		*	*	
Romanjuk.A (37)		*			*		*		
Jablonska.E (40)	*	*			*		*	*	
Quintana Pacheco. DA (38)	*	*	*	*	*			*	*

**Statistical analysis: **Studies were combined based on the sample size, mean and standard deviation. The formula of two integrated variance was used to calculate the difference between the average variance of the normal distribution. To assess heterogeneity between studies, the chi-square test and I^2^ index was used. In this study, the analysis was performed using a random-effects model, considering the significant heterogeneity between the studies. Integrated estimations and the related confidence interval of 95% were evaluated using forest plots as visuals. Sensitivity analyses were also performed. For evaluating publication bias, Funnel plots and Egger test were used. P<0.05 were considered as valid for heterogeneity tests. STATA (version 12) was used for statistical analyses.

## Results

In initial search process, 71 studies were identified. Of these studies, 9 duplicates were excluded. We excluded other 42 articles (review article, lack of enough information and comparison with other elements). After detailed review of selected articles, 20 published studies between 1984 and 2018 including four cross-sectional (20-22) and 17 case control (23-25), (26-30), (31-38) were selected for the final analysis (figure 1). Considering all the included studies, the total number of participants was 4,110 individuals containing 1,624 patients with breast cancer and 2,486 healthy subjects. Selected studies were conducted in different countries. Of the 20 studies, nine were conducted in Asia (25), (21, 22, 30-32), (34-36), six in Europe (20, 29, 39), (37, 38, 40), four in America (23, 24, 27, 34), and one were performed in Africa (18). Among the reviewed studies, in eight of them iron status was measured in serum and plasma (21, 25) (30, 32, 35, 36, 38, 39); in two studies scalp hair iron status was used (22, 31) and in the remaining ten breast tissues was the sample specimen used (18, 20, 23, 24, 27, 29, 33, 34, 37, 40). The quality of studies were assessed using the NOS; accordingly, three studies had a score of 7 , four studies had a score of 6), six studies had a score of 5 , three studies had a score of 4 and the remaining four studies had a score of 3. The baseline characteristics of these studies are summarized in table 2. 

**Figure 2:**
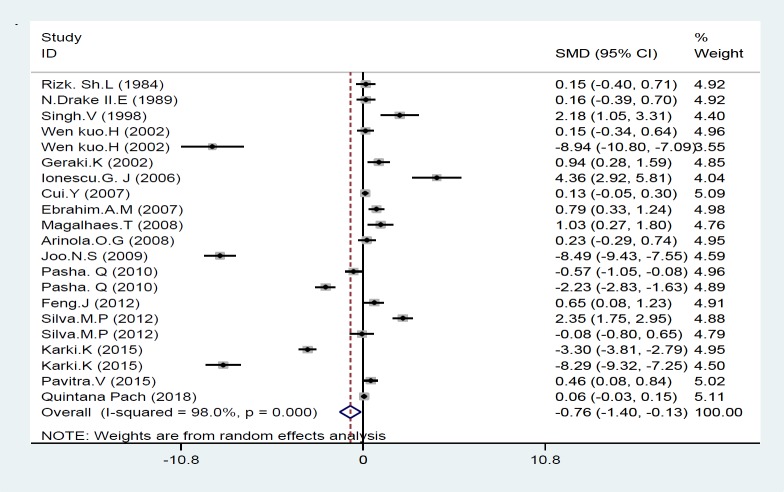
Forest plot of the association of iron with breast cancer risk. Square represents effect estimate of individual studies with their 95 % confidence intervals. And the diamond shows the overall estimate of SMD in this study. In this chart, studies are stored in order of the year of publication and author’s names, based on a random effects model

**Figure 3:**
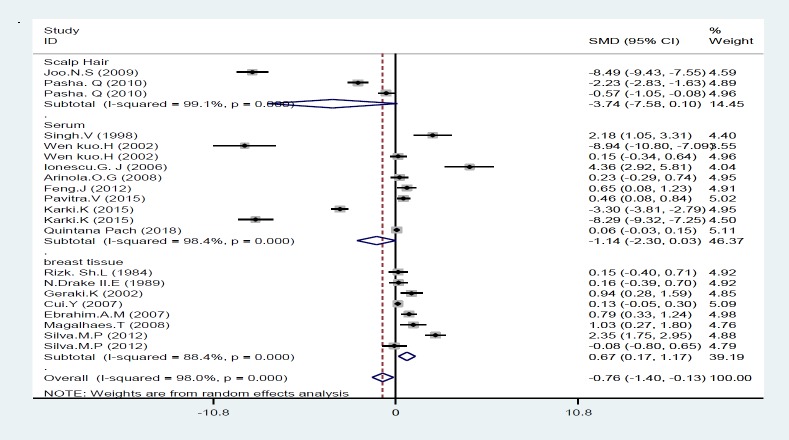
Forest plot of the association of iron with breast cancer risk based on sample type (serum and plasma, breast tissues, scalp hair)

In this study, the levels of iron in various studies in both the case and control groups were identified and standard mean difference (SMD) was measured in analysis. Among the included studies, two of them were excluded from analysis because they did not report the iron concentration in control group (34, 37). Also, one study presented data as mean and did not present standard deviation, so it was excluded from final analysis (40); overall, seventeen of 20 studies presented their findings as means±SD and were included in final analysis (table 2). As seen in figure 2, the present meta-analysis using a random effects model showed a significant association between Fe statuses and risk of breast cancer; the standard mean difference (SMD) was -0.76 [95% CI: -1.40 to -0.13; P<0.001] and there was a significant heterogeneity [I^2^=98.0%; P=0.000] (figure 2). We conducted a subgroup analysis according to the type of sample specimens; accordingly Fe concentrations were measured in three groups 1: serum and plasma, 2: breast tissue and 3: scalp hair. Our meta-analysis found a meaningful difference in Fe concentrations between individuals with and those without breast cancer only in breast tissue subgroup which SMD was 0.67 [95% CI: 0.17 to 1.17; P=0.009], whereas there was no meaningful difference in Fe statuses between women with and without breast cancer related to scalp hair and plasma subgroups; their mean difference was -3.74 [95% CI: -7.58 to 0.10; P=0.056] and -1.14[95% CI: -2.30 to 0.03; P=0.055], respectively (figure 3). 

**Table 2:** Baseline characteristics of studies included in this meta-analysis

**First author,** **(Reference)**	**Country (year of publication)**	**Case**	**Control**	**Matrix**	**Iron concentration (Mean±SD)**			Unit	**Type of measurement**
					**Case**		**Control**		
Rizk.Sh.L (23)	Chicago (1984)	25	25	Breast tissue	238.5±113		218.4±149.3	µg/g	EDXRF
N.Drake II.E(24)	Texas (1989)	26	26	Breast tissue	239±113		218±149	µg/g	INAA
Singh.V (25)	India (1998)	10	10	Serum	2250±350		1710±7	µg/g	AAS
Geraki.K (20)	UK (2002)	20	20	Breast tissue	16.40±14.11		6.06±6.57	ppm	TXRF
Wen Kuo.HW (21)	China (2002)	43	26	Serum Benign	1055.81±104.05		1040.38±99.94	µg/l	ICP-AES
Wen Kuo.HW (21)	China (2002)	43	25	Serum Malignant	114.71±107.12		1040.38±99.94	µg/l	ICP-AES
Ionescu.J.G (39)	Prague, Czech Republic, Germany (2006)	20	8	Plasma	53.17±10.20		10.94±8.10	µg/kg	AAS- ICP-MS
Cui.Y (27)	USA (2007)	251	249	Breast tissue	2.38±2.10		2.12±2	ng/cm^2^	TXRF
Ebrahim.A.M(18)	Sudan (2007)	40	40	Breast tissue	66.10±12.79		57.92±7.23	ppm	INAA
Magalhaes.T (29)	Portugal, Germany (2008)	15	15	Breast tissue	43.20±9.8		34±7.90	µg/g	TXRF
Arinola, O. G (30)	Nigeria(2008)	29	30	Plasma	59.62±2.53		59.05±2.50	µg/l	AAS
Joo,N (22)	South Korea (2009)	40	144	Hair	6.45±0.43		9.15±0.28	µg/g	-
Pasha. Q (31)	Pakistan (2010)	33	35	Scalp Hair Malignant	114±24.50		129±28.10	µg/g	AAS
Pasha. Q (31)	Pakistan (2010)	36	35	Scalp Hair Benign	80.40±13		129±28.10	µg/g	AAS
Feng.J.F (32)	China (2012)	32	20	Serum	1146.30±156.2		1062.3±59.2	µg/l	M6 AAS
Silva.M (33)	Brazil (2012)	34	38	Breast tissue Malignant	33±8.30		15.60±6.50	mg/kg	TXRF
Silva.M (33)	Brazil (2012)	9	38	Breast tissue Benign	15.1±6.30		15.60±6.50	mg/kg	
Rehman.S (34)	Pakistan (2014)	15	-	Breast tissue Benign	49.1±11.4		-	mg/l	AAS
Rehman.S (34)	Pakistan (2014)	20	-	Breast tissue Malignant	225±121		-	mg/l	AAS
Pavithra.V(35)	India (2015)	54	54	Serum	85.47±47.45		67.49±28.24	-	Ferrozo
Karki. K (36)	India (2015)	70	70	Serum Benign	57.26 ±3.69		69.33±3.62	µg/l	ferrozine
Karki. K (36)	India (2015)	70	70	Serum Malignant	46.73±1.32		69.33±3.62	µg/l	ferrozine
Romanjuk.A (37)	Ukraine (2016)	20	-	Breast tissue	2.0 ±0.26		-	g/mol	EDXRF
Jablonska.E (40)	Poland (2017)	42	42	Breast tissue	66.50		41.20	µg/g	AAS
Quintana Pacheco. DA (38)	Germany (2018)	627	1466	Plasma	17.80±6.60		17.4±6.60	µmol/l	The Roche Cobas 6000 analytical system

**Abbreviations: **Atomic absorption spectrophotometry (AAS); inductively coupled plasma–atomic emission spectrometry (ICP-AES); X-ray fluorescence spectrometry (TXRF); Instrumental neutron activation analysis (INAA); Energy dispersive X-ray fluorescence (EDXRF).

**Figure 4:**
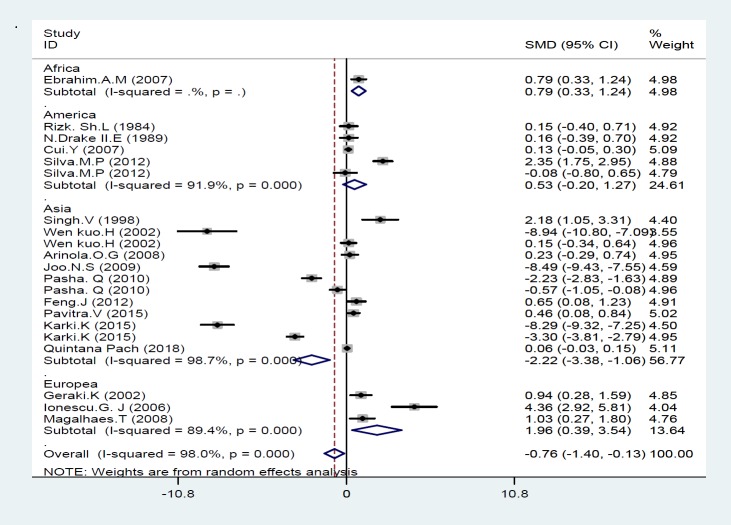
Forest Plot of the association of iron with breast cancer risk based on continent

Figure 4 shows the result of meta-analysis for each continent. As seen, there was significant statistical difference in Fe concentration between cancer patients and healthy controls in Asia, Europe and Africa; their SMD was -2.22 [95% CI: -3.38 to -1.06; P=0.000], 1.96 [95% CI: 0.39 to 3.53; P=0.014] and 0.79 [95% CI: 0.33 to 1.24; P=0.001], while no significant difference was observed in Fe concentration between cancer patients and healthy women in America; the mean difference was 0.53 [95% CI: -0.2 to 1.27; P=0.154]. We performed a subgroup analysis based on the quality score; the results of meta-analysis showed a meaningful difference in Fe status between breast cancer and control group only in high quality studies (≥7 stars) which the mean difference was -4.19 [95% CI: -7.27 to -1.10; P=0.00], suggested that Fe status was associated with an increased risk of breast cancer: Only in studies with high quality score. 

**Figure 5:**
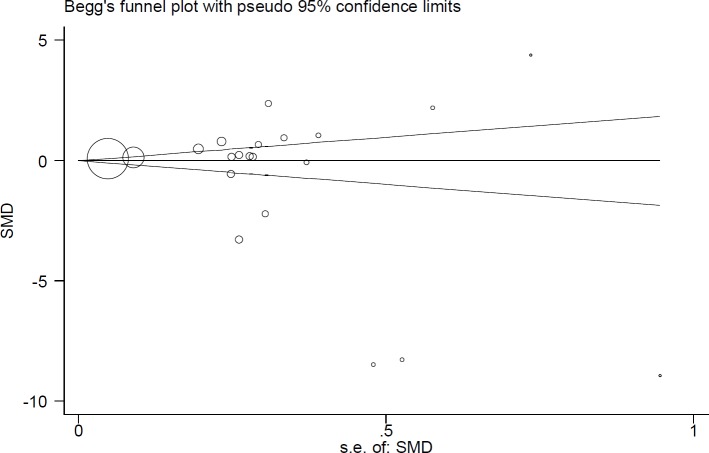
Begg’s funnel plot for publication bias

Figure 5 presents the Beggs funnel plot of tests related to Fe statuses in cancer patients. Interpretation of this plot showed no signs of publication bias in these studies (p=0.24); this means that studies with negative and positive results have been published (figure 5).

## Discussion

This study systematically reviewed the effects of iron in breast cancer and quantitatively analyze the association between iron levels and the risk of breast cancer. The current review including 20 studies (figure 2), showed that high levels of iron have significant relationship with increased risk of breast cancer (P=0.053). In this regard, serum, breast tissues and scalp hair status of iron were examined. We observed a meaningful association between iron levels in breast tissues and an increased risk of breast cancer (p≤0.05). Whereas, analysis of Fe concentrations in serum and scalp hair did not reveal any significant relationship between iron statuses and the risk of breast cancer (p>0.05). So far, some studies have evaluated the relationship between iron concentration and the risk of breast cancer and have reported contradictory results. Our findings were consistent with previous studies as demonstrated iron status can increase the incidence of breast cancer (18, 20-23, 29, 31-33, 35, 36, 39); higher levels of iron were detected in breast tumor tissues relative to the normal tissues in some studies (41, 42), also higher concentrations of serum iron was observed in women with breast tumor compared to healthy subjects (43, 44). Whereas others did not report a positive link between Fe concentration and breast cancer (24, 25, 27, 30, 34, 37).

The present study via meta-analysis method proved a direct relationship between high iron levels and greater risk of breast cancer. Similarly, a systematic review summarized the evidence regarding the relationship between Fe and breast cancer risk and concluded that this trace element can apply a stimulatory function in breast cancer (10). Accordingly, it was established that higher iron concentrations can enhance risk of breast cancer in women (41-44). However, iron abundantly exists in all human organisms and performs many functions. Overload iron may have toxic effects; for example, it contributes in generating free radicals, a reaction that is regulated by antioxidant mechanisms whenever iron concentration is in a normal state (15, 17-19).

Iron is necessary for physiological cellular functions because it is an inseparable part of many proteins and enzymes (45). It fundamentally contributes in the arrangement of cellular growth and differentiation (46). Physiological keeping of fairly stable iron levels is a vital condition so, both insufficiency and excess intake of iron have negative effects and can lead to disease expansion (15). High iron load is related to many chronic conditions, like imperfect control of the immune system (44) and cancer (45, 46). Iron can be carcinogenic. Several studies evaluated the relationship between elevated iron levels and risk of cancer; some of these studies reported that iron overload was linked to greater risk of overall cancer and cancer mortality (43, 47), while others did not observe this association (48). Iron overload is able to contribute in the generation of reactive oxygen species, involve in the repress of cellular immune functions, and raise tumor growth (49). Oxidative and reduction reactions involving in Fe storage and transport exhibits that it involved in the generation of free radical according to the equations that is known as Fenton and Haber-Weiss reactions (41): Fe^2+^ + H2O2 Fe^3+ ^+ OH + OH ^–^

These reactions produce hydroxyl radicals that lead to lipid peroxidation, DNA damage, inactivate enzymes, and depolymerize polysaccharides and apoptosis (49-51). In breast cancer patients, free iron may be released from the storage form during the metabolism of estradiol, this reaction induces oxidative stress that leads to the generation of mutagenic radicals, and then the produced free radicals cause DNA damage and mutations in the breast (12, 48). It is accepted that the expression of several iron-regulatory proteins such as ferritin, hepcidin and ferroportin are deregulated in breast cancer subjects (41). Thus, high concentration of iron is considered as a major risk factor in the progression of breast cancer. 

Iron as a nutritious substance participates in the feeding of microbial and neoplastic cells and thereby leading to disease progress (52). Iron also plays an important role in simulating angiogenesis (53). Bio activation of phenolic/quinonic compounds near the tumor location can abundantly produce the radicals of superoxide and semiquinone which have detrimental effects on the metal-rich cancer cells (54, 55). This causes inflammation and increased growth of cancer cells (56). Some studies have reported that excessive agglomeration of iron in humans is related to a high risk of cancer (57-59). However, others have reported conflicting results and not found any significant relationship between accumulation of iron and breast cancer risk (60, 61).

We conducted subgroup analysis based on the type of sample specimens to address the observed heterogeneity, and we could not find a meaningful association between iron levels in serum and scalp hair with breast cancer, whereas our analysis revealed a significant relationship between high levels of iron and greater risk of breast cancer in breast tissue and also a relatively mild association in benign breast tissue. One possible reason for this observation is that excessive accumulation of free iron in benign breast tissue due to their catalytic effects can generate mutagenic radicals, and also can repress host defense cells and, thus enhances the risk of breast cancer (28).

One of the other possible reasons is that both benign and cancerous cells may request enhanced concentration of iron for maintaining their proliferation due to iron is essential for ribonucleotide reductase which is a main enzyme in DNA synthesis (62). Therefore, it is likely that benign breast tissue with high iron accumulation be prone to breast cancer. 

We also conducted subgroup analysis based on geographic region; analysis of the association Fe statuses with breast cancer based on continent showed a significantly difference in Fe concentration between breast cancer and healthy women in Asia, Europe and Africa (p<0.05); while, no meaningful difference was found in Fe concentration between women with and without breast cancer in America (P>0.05). In Asia, low levels of Fe and in Europe and Africa high levels of Fe had positive association with the risk of breast cancer. Geographical and residential socioeconomic inequalities may potentially reflect the observed differences. Also, demographic characteristics and genetic background of different populations can also explain the observed heterogeneity. In addition, our analysis based on the quality score showed a meaningful correlation between Fe statuses and breast cancer risk only in high quality studies that this may also affect the results of heterogeneity.

It has been hypothesized that dietary iron intake was positively associated with an increased breast cancer risk and several studies were performed in this regard; some of them found null results (63-65). Whereas several large prospective cohort studies reported a positive association between iron intake and the risk of breast cancer (66-68). These findings propose that higher iron intake for example what is found at red meat may enhance the breast cancer risk. Along with confirming this, the current study showed a high concentration of iron in human is positively associated with an enhanced risk of breast cancer. Thus, high iron accumulation in the body according to their effect in the progression of breast cancer is important among women, especially those who are prone to developing cancer. Since, deregulation of iron metabolism related biomolecules is one of the most important factors for breast cancer, cancer can be prevented through regulating iron metabolism related proteins and prevention of iron accumulation in the tissue. This information can be used by researchers to manufacture drugs that can control the pathways related to iron accumulation and carcinogenesis. Besides, the study about the association between iron and breast cancer risk and burden of disease provides a unique perspective for planning interventions and developing public health policies. Moreover, our findings indicated that preventive procedures and interventional methods for controlling concentration of iron in human are needed especially in those who are prone to developing cancer. 

## Limitations

This meta-analysis had several limitations that most of them were related to methodology. Some of these limitations include:

1. Absence of the same method for measuring variance.

2. Absence of data relation to habits and lifestyle of the population studied.

4. The screening methods varied and there was not a uniform standard method for measuring iron concentrations in different studies.

5. Some relevant articles were not available 

In conclusion the present meta-analysis indicated a positive and straight association between iron concentration and risk of breast cancer (P=0.009). But because of high heterogeneity we recommend more accurate future studies.

Thus, Fe levels should be controlled in food sources, drugs, and etc. This study showed that the estimation of iron concentration in biological specimens has an important role in early detection of this disease. Our results also can be considered for debarment of breast cancer by controlling level of iron in the persons' diet. 

## Financial Disclosure:

There are no benefits in any form that have been or will be received from a commercial party related directly or indirectly to the subject of this article.
